# Serum biomarkers to predict risk of testicular and penile cancer in AMORIS

**DOI:** 10.3332/ecancer.2017.762

**Published:** 2017-08-23

**Authors:** Arunangshu Ghoshal, Hans Garmo, Rhonda Arthur, Niklas Hammar, Ingmar Jungner, Håkan Malmström, Mats Lambe, Göran Walldius, Mieke Van Hemelrijck

**Affiliations:** 1 King’s College London, Division of Cancer Studies, Translational Oncology and Urology Research, London SE1 9RT, UK; 2 Regional Cancer Centre, Uppsala University, Box 256 751 05, Uppsala, Sweden; 3 Unit of Epidemiology, Institute of Environmental Medicine, Karolinska Institutet, Stockholm SE-171 77, Sweden; 4 AstraZeneca R and D, Mölndal 431 50, Sweden; 5 Department of Medicine, Clinical Epidemiological Unit, Karolinska Institutet and CALAB Research, Stockholm SE-171 77, Sweden; 6 Department of Medical Epidemiology and Biostatistics, Karolinska Institutet, Stockholm SE-171 77, Sweden; 7 Unit of Cardiovascular Epidemiology, Institute of Environmental Medicine, Karolinska institutet SE-171 77, Stockholm, Sweden; 8 Department of Palliative Medicine, Tata Memorial Hospital, Mumbai, Maharashtra 400012, India

**Keywords:** penile cancer, testicular cancer, serum inflammatory markers, albumin, cohort study, survival analysis

## Abstract

**Purpose:**

To evaluate the association between commonly measured serum biomarkers of inflammation and penile and testicular cancer risk in the Swedish Apolipoprotein-related MORtality RISk (AMORIS) study.

**Materials and methods:**

A total of 205,717 subjects had baseline measurements of C-reactive protein, albumin, and haptoglobin. The association between quartiles and dichotomised values of inflammatory markers and penile and testicular cancer risk were analysed by using multivariate Cox proportional hazard models.

**Results:**

A total of 125 men were diagnosed with testicular cancer and 50 with penile cancer during a mean follow-up of 20.3 years. No statistically significant trends were seen between serum inflammatory markers and risk of penile cancer, but higher albumin levels increased the risk of testicular cancer [HR for albumin (g/L): 1.10 (95% CI: 1.03–1.18)]. However, this trend was not observed when using medical cut-offs of albumin.

**Conclusions:**

In the present study, we did not find support for an association between commonly used markers of inflammation and risk of testicular or penile cancer. The role of inflammation may be more complicated and require assessment of more specialised measurements of inflammation in future studies.

## Introduction

About 2300 men are diagnosed with testicular cancer in the United Kingdom each year. More than half of these men are younger than 35 years. About 600 men are diagnosed with penile cancer (CRUK) [1]. Even though these cancers are less common, there is a need for further research into their potential risk factors as both diseases are associated with a significant reduction of quality of life [2]. Second primary tumours, cardiovascular disease, neurotoxicity and ototoxicity, pulmonary complications, hypogonadism, and nephrotoxicity are some of the potentially life-threatening long-term complications of testicular cancer and its therapy, whereas urethral stenosis, necrosis, reduced voiding ability and sexual function are some of the factors reducing quality of life in men treated for penile cancer [3].

Little is known about the risk factors for these rather uncommon cancers. Chronic inflammation is thought to initiate or promote cancer through generation of reactive oxygen species and proinflammatory cytokines; and conversely, inflammation may occur secondary to cancer and affect disease progression [4]. In this context, many studies have looked into the more common cancers like breast and prostate [5]. However, few studies have focused on the role of inflammation in risk of testicular or penila cancer. Studies done on testicular germ cell neoplasia have shown that B cells and supporting cytokines, such as IL-6 and CXCL-13, are involved in the immunopathology. The pro-inflammatory, pro-tumorigenic cytokine environment is likely to facilitate tumour progression and invasion [6]. A Norwegian cohort study of 586 men with testicular cancer also observed an association between serum levels of C-reactive protein (CRP) and development of second primary tumours and cardiovascular disease [7]. In a case–control study with 36 patients with localised testicular cancer, it was found that leucocyte counts and neutrophil to lymphocyte ratio were significantly higher in patients with testicular cancer compared with the control group [8]. With respect to penile cancer, chronic inflammation caused by persistent human papillomavirus infection has been linked with risk of penile cancer as autocrine and paracrine signals are thought to mediate oncogenic action, causing changes in somatic cells under the influence of the microbial genome or of epigenetic factors [9]. A German study based on 79 cases found that high pre-operative levels of serum CRP were associated with poor survival [10]. In a retrospective study, high pre-surgical CRP levels were significantly associated with lymph nodal involvement and poor outcomes [11].

Overall, most of studies looking into biomarkers of inflammation and risk of testicular or penile cancer were limited due to small sample size and focused on cancer prognosis and quality of life [3], rather than risks of developing these cancers. Hence, the current study aims to evaluate the association between serum markers of chronic inflammation and risk of developing testicular or penile cancer.

## Materials and methods

### Study population and data collection

The Swedish Apolipoprotein-related MORtality RISk database (AMORIS) includes blood samples from 812,073 Swedish men and women, ranging in age from < 20 to 80 years old and over undergoing occupational health screening or primary care. The cohort is based on a linkage between data from laboratory examinations performed in the Central Automation Laboratory (CALAB) in Sweden and information recorded in Swedish National Registers using a 10-digit personal identifier number, which is unique to every Swedish resident [12]. AMORIS is a large prospective cohort with information on serum biomarkers, cancer diagnosis, comorbidities, vital status, socio-economic status and emigration. This study complied with the Declaration of Helsinki and was approved by the Ethics Review Board of the Karolinska Institute.

We restricted our study population to individuals aged 20 years or older who did not have a previous diagnosis of cancer. Furthermore, all subjects were required to have baseline measurements of CRP, albumin, haptoglobin available from the same health examination (*n* = 205,717). The outcome investigated in this study was penile cancer (International Classification of Diseases (ICD), Revision 7 (1955) code 179) and testicular cancers (ICD Revision 7 (1955) code 178) [13].

Serum CRP (mg/L), albumin (g/L), haptoglobin (g/L), triglycerides (mmol/L), total cholesterol (mmol/L), glucose (mmol/L), uric acid and creatinine were measured at baseline with standard laboratory methods as described elsewhere [14]. High-sensitive CRP (hsCRP) was not available at any time in the period of blood sample collection (1985–1996), so that CRP concentrations < 10 mg/L could not be measured precisely [15]. However, the cut-off point of 10 mg/L is widely accepted as the upper limit of the health-associated reference range and was therefore used in this study [16]. Levels of serum inflammatory markers were assessed as high or low based on their clinical cut-offs used in CALAB: CRP 10 mg/L and haptoglobin 1.4 g/L. For albumin, a cut-off point of 40 g/L was used instead of 35 g/L due to the small number of participants with low albumin levels.

Serum glucose, total cholesterol, and triglycerides levels were dichotomised using clinical cut-offs in accordance with the American Diabetes Association and National Cholesterol Education Programme (NCEP) guidelines (cut-offs: 6.11, 6.50 and 1.71 mmol/L for glucose, total cholesterol and triglycerides, respectively) [17]. In addition, the following baseline information was obtained from AMORIS: educational level (low, intermediate, high), Charlson comorbidity Index (CCI; 0, 1, 2, ≥ 3) and fasting status (fasting, non-fasting, missing). Follow-up time was defined as the time from baseline measurements until date of cancer diagnosis, date of death, emigration or end of study (31st December 2011), whichever came first.

### Data analyses

We estimated the risk of penile and testicular cancer with multivariate Cox proportional hazards regression, comparing people with high to low levels of CRP, albumin and haptoglobin, respectively. Cox regression models were adjusted for age, sex, education and CCI, as well as triglycerides (continuous), glucose (continuous), total cholesterol (continuous), fasting status, uric acid (continuous) and creatinine (continuous). The assumption of proportional hazards was evaluated by adding time-dependent covariates into the models and assessment of the Schoenfeld Residuals.

Based on current evidence, the World Trade Center Program Administrator under Centers for Disease Control and Prevention (CDC) sets the minimum latency for thyroid cancer as 2.5 years, which is well covered within our follow-up time [18]. Nevertheless, we assessed reverse causality through two sensitivity analyses excluding those with follow-up time < 3 years and < 5 years, respectively.

For those biomarkers where we observed an association based on the hazard ratios, we used the restrictive cubic spline (RCS) function to graphically display the hazard ratios representing the dose–response association. We used knots located at the 5th, 25th, 75th, and 95th percentiles as well as the medical reference value [19] in a multivariate Cox proportional hazards model as described above. This analysis was performed using the RCS_Reg SAS Macro created by Desquilbet and Mariotti [20]. All analyses were conducted with Statistical Analysis Systems (SAS) software release 9.4 (SAS Institute, Cary, NC) [21].

## Results

Characteristics of study participants are shown in Table 1. A total of 125 men developed testicular cancer and 50 men developed penile cancer during a mean follow-up of 8.9 years and 13.3 years, respectively (20.3 years in the cohort who did not develop penile or testicular cancer). No statistically significant trends were seen between the included serum inflammatory markers and risk of penile cancer, but higher albumin levels were associated with an increased risk of testicular cancer [HR for albumin (g/L): 1.10 (95% CI: 1.03–1.18)]. However, this trend was not observed when using medical cut-offs of albumin (Table 2).

**Figure 1.**
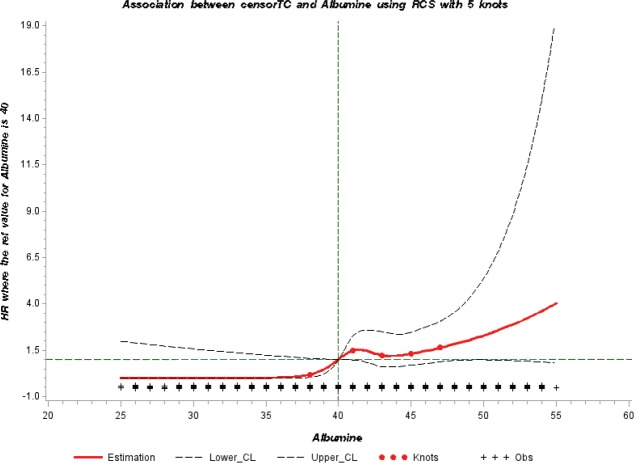
Dose response relationship between serum albumin levels and HR of testicular cancer by RCS curve with 5 knots of albumin values at 5th, 25th, 50th, 75th, 95th percentiles [Reference value of albumin = 40 g/L]

To further evaluate the potential association between serum albumin and risk of testicular cancer, we modelled a dose–response curve with restrictive cubic splines (Figure 1). The shape of the curve was consistent with the direction of the hazard ratios observed. A sensitivity analysis to assess reverse causation by excluding those with follow-up time < 3 years and < 5 years did not affect the above findings (results not shown).

## Discussion

In the present study, we found that higher serum albumin levels are associated with higher risks of testicular cancer in the AMORIS cohort, but no association was observed between serum levels of CRP or haptoglobin and risks of penile or testicular cancer.

Most of the studies on testicular cancer have focussed on tumour immunity and have shown that B cells and B-cell-supporting cytokines, such as IL-6 and CXCL-13, are involved in the immunopathology of testicular germ cell neoplasia. In those studies, it has been emphasised that the pro-inflammatory, pro-tumorigenic cytokine environment likely facilitates tumour progression and invasion [6]. In the case of penile cancer, studies have mostly focussed on viral aetiology of carcinogenesis like in the case of human papillomavirus (HPV)–induced penile cancers. They have stressed on the associations between chronic inflammation, persistent infection, and cancer, where autocrine and paracrine signals mediate oncogenic action, causing changes in somatic cells under the influence of the microbial genome or of epigenetic factors [9]. None of the studies have considered systemic markers of inflammation and carcinogenesis. Most of the studies investigating serological biomarkers in context of penile [10, 11] and testicular cancers [8, 22–24] have investigated on disease diagnosis, progression, treatment response, and late side effects with little focus on risks.

In our study, analysis of CRP and haptoglobin in relation to risks of these cancers have showed no association, but serum levels of albumin were found to be positively related to the risk of subsequent development of testicular cancer. The observed association slightly weakened when the model was adjusted for potential confounders. These findings suggest the importance of commonly measured biomarker like albumin to predict the risks of testicular cancer patients. However, detailed explanation for the observed association could not be generated, as it was outside the scope of this project and needs further basic research. To date, this is the largest prospective study assessing common serum inflammatory markers in relation to these cancers. Nevertheless, there were some limitations that need careful acknowledgement.

The strength of studies conducted within AMORIS lies in the prospective evaluation of exposures and complete follow-up of study participants. All analyses were performed at the same laboratory with internationally accredited and calibrated methods [14]. The population in AMORIS was selected by analysing fresh blood samples from health check-ups in non-hospitalised persons; however, possibility of batch effects, human error in data imputation, and missing data cannot be ruled out [25]. Any healthy worker effect would not influence the internal validity of the conducted studies. Information on race/ethnicity was not available, but the AMORIS cohort was like the general working population of Stockholm [26], which comprised about 90% in 1990 and 80% Swedish-born individuals in the year 2000 [27].

It is a limitation that we did not have high-sensitive CRP available at the time measurements were conducted. Any CRP levels < 10 mg/L were unquantifiable, which may have resulted in an underestimation of the association between serum CRP and these cancers. Information on BMI was only available for a small proportion of the participants in the current subset of AMORIS participants. There was limited information for other potential confounders such as smoking habits, history of HPV infection, tumour histology or other serum inflammatory markers such as IL-6 or IL-8. However, all models were adjusted for Charlson Comorbidity Index. The different histological variants of penile or testicular cancers might have different courses in their natural history of carcinogenesis [28, 29], but that could not be verified in the present study.

## Conclusion

In the present study, we did not find support for an association between commonly used markers of inflammation and risk of testicular or penile cancer. Future studies should investigate temporality of associations between inflammation and these cancers using a modelling approach based on repeated measurement of time-to-event data with more detailed markers of systemic inflammation to provide more insight into these possible links.

## List of abbreviations

AMORIS: Apolipoprotein MORtality RISk
CALAB: Central Automation Laboratory
CCI: Charlson Comorbidity Index
CDC: Centers for Disease Control and Prevention
CI: Confidence interval
CRP: C-reactive protein
CRUK: Cancer Research United Kingdom
CXCL-13: CXCL-13
g/L: gram per litre
G-CSF: Granulocyte-colony stimulating factor
HPV: Human papilloma virus
HR: Hazard ratio
hsCRP: High sensitive CRP
IL-6: Interleukin 6
IL-8: Interleukin 8
mg/dl: milligram per decilitre
Mmol/L: millimoles per litre
NCEP: National Cholesterol Education Programme
RCS: restricted cubic splines
SAS: Statistical Analysis System
SD: Standard deviation

## Compliance with ethical standards

### Funding

The research was funded/supported by the Swedish Cancer Society, grants from the Gunnar and Ingmar Jungner Foundation for Laboratory Medicine, Stockholm, Sweden, the National Institute for Health Research (NIHR) Biomedical Research Centre based at Guy’s and St Thomas’ NHS Foundation Trust and King’s College London. The views expressed are those of the author(s) and not necessarily those of the Cancerfonden, NHS, the NIHR or the Department of Health.

## Conflicts of interest

The authors declare that they have no conflict of interest.

## Authors’ contributions

A Ghoshal and H Garmo performed project development, data analysis, manuscript writing. Data analysis and manuscript editing are done by R. Arthur. Data collection and manuscript editing are done by N Hammar, I Jungner, H Malmstrom, M Lambe, and G Walldius. Project development and manuscript editing are done by M Van Hemelrijck.

## Figures and Tables

**Table 1.** Descriptive statistics of the study population by cancer status

	All men (*n* = 205,717)		
	Penile cancer (*n* = 50)	Testicular cancer (*n* = 125)	No cancer (*n* = 205,542)
**Mean age (yrs.) (SD)**	51.0 (10.6)	35.3 (10.3)	43.8 (11.5)
**Socio-economic status (%)**			
White collar	32 (64.00)	65 (52.00)	103,405 (50.31)
Blue collar	17 (34.00)	50 (40.00)	93,075 (45.28)
Unemployed/missing	1 (2.00)	10 (8.00)	9062 (4.41)
**Education (%)**			
Low	19 (38.00)	24 (19.20)	52,449 (25.52)
Middle	20 (40.00)	60 (48.00)	91,095 (44.32)
High	11 (22.00)	41 (32.80)	57,891 (28.16)
Missing	0 (0.00)	0 (0.00)	4107 (1.99)
**Charslon Comorbidity Index (%)**			
0	45 (90.00)	120 (96.00)	193,191 (93.99)
1	4 (8.00)	3 (2.40)	6926 (3.37)
2	1 (2.00)	2 (1.60)	4148 (2.02)
3+	0 (0.00)	0 (0.00)	1277 (0.62)
**Body mass index (kg/m^2^) (%)**			
< 18.5	0 (0.00)	0 (0.00)	773 (0.37)
18.5–24.99	8 (16.00)	20 (16.00)	26,570 (12.93)
25–29.99	5 (10.00)	8 (6.40)	14,298 (6.96)
≥ 30	3 (6.00)	1 (0.80)	3483 (1.69)
Missing	34 (68.00)	96 (76.80)	160,418 (78.05)
**CRP (mg/L) (%)**			
Mean (SD)	3.90 (1.0)	7.69 (1.0)	5.41 (3.0)
< 10	44 (88.00)	102 (81.60)	170,859 (83.13)
≥ 10	6 (12.00)	23 (18.40)	34,683 (16.87)
**Albumin (g/L) (%)**			
Mean (SD)	42.42 (43.0)	44.41 (44.0)	42.92 (43.0)
< 40	7 (14.00)	3 (2.40)	20,838 (10.14)
≥ 40	43 (86.00)	122 (97.60)	184,704 (89.86)
**Haptoglobin (g/L) (%)**			
Mean (SD)	1.11 (1.10)	0.98 (1.0)	1.04 (1.0)
< 1.4	44 (88.00)	112 (89.60)	178,771 (86.97)
≥ 1.4	6 (12.00)	13 (10.40)	26,771 (13.02)
**Fasting status (%)**			
Fasting	32 (64.00)	69 (55.20)	127,396 (61.98)
Not fasting	18 (36.00)	56 (44.80)	76,540 (37.24)
Missing	0 (0.00)	0 (0.00)	1606 (0.78)
**Fasting glucose (mmol/L) (%)**			
Mean (SD)	5.10 (0.75)	4.86 (0.84)	4.90 (1.21)
< 6.11	46 (92.00)	116 (92.80)	193,211 (94.0)
≥ 6.11	4 (8.00)	9 (7.20)	10,725 (5.22)
Missing	0 (0.00)	0 (0.00)	1606 (0.78)
**Triglycerides (mmol/L) (%)**			
Mean (SD)	1.76 (0.96)	1.21 (0.79)	1.31 (1.06)
< 1.71	27 (55.10)	103 (82.40)	162,839 (79.22)
>=1.71	22 (44.90)	22 (17.60)	41,189 (20.04)
Missing	1 (0.02)	0 (0.00)	1514 (0.74)
**Total cholesterol (mmol/L) (%)**			
Mean (SD)	5.75 (0.96)	5.15 (1.09)	5.54 (1.12)
< 6.5	38 (76.00)	113 (90.40)	164,141 (79.86)
≥ 6.5	12 (24.00)	12 (9.60)	39,894 (19.41)
Missing	0 (0.00)	0 (0.00)	1507 (0.73)
**Uric acid (%)**			
Mean (SD)	325.68 (55.19)	305.68 (55.16)	284.61 (71.12)
Missing	0 (0.0)	1 (0.8)	1967 (0.96)
**Creatinine (%)**			
Mean (SD)	89.36 (11.75)	86.18 (10.27)	80.26 (14.16)
Missing	0 (0.0)	1 (0.8)	1458 (0.71)

**Table 2.** Hazard ratio (HR) for risk of testicular and penile cancer, with 95% confidence intervals (CI) from Cox proportional hazards model.

Penile cancer (n = 50)				Testicular cancer (n = 125)		
	Crude	Adjusted^1^	Adjusted^2^	Crude	Adjusted^1^	Adjusted^2^
	HR (95% CI)	HR (95% CI)	HR (95% CI)	HR (95% CI)	HR (95% CI)	HR (95% CI)
**CRP (mg/L)**						
Continuous	0.97 (0.91–1.03)	0.96 (0.9–1.03)	0.95 (0.89–1.02)	1.00 (0.99–1.01)	1.00 (0.99–1.01)	1.00 (0.99–1.01)
≥ 10	0.59 (0.25–1.41)	0.55 (0.23–1.30)	0.52 (0.22–1.24)	1.07 (0.68–1.68)	1.13 (0.71–1.78)	1.03 (0.65–1.65)
**Albumin (g/L)**						
Continuous	0.93 (0.84–1.03)	1.76 (0.50–6.16)	0.98 (0.88–1.10)	**1.21 (1.14–1.29)**	**1.13 (1.06–1.21)**	**1.10 (1.03–1.18)**
≤ 40	1.52 (0.68–3.37)	1.14 (0.51–2.56)	1.20 (0.53–2.72)	**0.22 (0.07–0.71)**	**0.29 (0.09–0.93)**	0.35 (0.11–1.09)
**Haptoglobin (g/L)**						
Continuous	1.99 (0.91–4.34)	1.43 (0.62–3.31)	1.20 (0.51–2.84)	0.49 (0.26–0.96)	0.79 (0.41–1.54)	0.68 (0.35–1.32)
≥ 1.4	0.97 (0.41–2.28)	0.78 (0.33–1.84)	0.68 (0.29–1.61)	0.81 (0.46–1.44)	1.07 (0.59–1.91)	0.89 (0.49–1.64)

1 adjusted for age, sex, education, Charslon Comorbidity Index.

2 adjusted for age, sex, education, Charslon Comorbidity Index, triglyceride (continuous), glucose (continuous), total cholesterol (continuous), fasting status, urea (continuous), creatinine (continuous).
